# Rhodopsin and melanopsin coexist in mammalian sperm cells and activate different signaling pathways for thermotaxis

**DOI:** 10.1038/s41598-019-56846-5

**Published:** 2020-01-10

**Authors:** Debarun Roy, Kohava Levi, Vladimir Kiss, Reinat Nevo, Michael Eisenbach

**Affiliations:** Department of Biomolecular Sciences, The Weizmann Institute of Science, 7610001 Rehovot, Israel

**Keywords:** Cellular imaging, Cell signalling, Chemotaxis

## Abstract

Recently, various opsin types, known to be involved in vision, were demonstrated to be present in human and mouse sperm cells and to be involved there in thermosensing for thermotaxis. In vision, each opsin type is restricted to specific cells. The situation in this respect in sperm cells is not known. It is also not known whether or not both signaling pathways, found to function in sperm thermotaxis, are each activated by specific opsins, as in vision. Here we addressed these questions. Choosing rhodopsin and melanopsin as test cases and employing immunocytochemical analysis with antibodies against these opsins, we found that the majority of sperm cells were stained by both antibodies, indicating that most of the cells contained both opsins. By employing mutant mouse sperm cells that do not express melanopsin combined with specific signaling inhibitors, we furthermore demonstrated that rhodopsin and melanopsin each activates a different pathway. Thus, in mammalian sperm thermotaxis, as in vision, rhodopsin and melanopsin each triggers a different signaling pathway but, unlike in vision, both opsin types coexist in the same sperm cells.

## Introduction

Sperm thermotaxis has been demonstrated in humans, rabbits and mice^1–4^. In this process, sperm cells swim to a warmer temperature by actively changing their swimming direction according to the temperature gradient^1,5^. The changes in swimming direction are mainly done by modulating the frequency of turns and hyperactivation events^6^. Not every sperm cell can respond by thermotaxis to the temperature gradient. Only capacitated cells do, i.e., cells that acquired a state of ripening, which confers on them the ability to fertilize the oocyte^7,8^. Since the process of capacitation (ripening) occurs asynchronously, only up to ~10% of the sperm population are capacitated at any given moment^9^ and, therefore, only up to ~10% are thermotactically responsive^1^. Hitherto, due to obvious reasons, sperm thermotaxis has only been demonstrated *in vitro*^1,5^. However, the finding that a temperature gradient is generated at ovulation within the oviduct^10–12^ strongly suggested that sperm thermotaxis also occurs *in vivo*^12^ and acts there as a long-range guidance mechanism^13,14^. As a matter of fact, sperm thermotaxis was recently even employed for selecting high-quality cells for intracellular sperm insemination (ICSI), resulting in large improvement of the ICSI outcome^15^.

The thermosensors for sperm thermotaxis in mammals are opsins^4^. These are G-protein-coupled receptors known to act as photosensors in vision. In mammalian eyes, at least nine opsin types are known^16^. Each of them is restricted to a specific cell type. For example, rhodopsin (Opsin-2) is present in rod cells^17^; blue opsin (Short-wave-sensitive opsin 1), green opsin (Medium-wave-sensitive opsin 1), and red opsin (Long-wave-sensitive opsin 1) are present in cone cells^18^; melanopsin (Opsin-4) is located in retinal ganglion cells^19^; and encephalopsin (Opsin-3) and neuropsin (Opsin-5) exist in retinal pigmented epithelium of the inner retina^20^. Opsins are also present in organs other than eyes, including the brain, lungs, liver, kidneys and skin^21–23^, but their functions in these organs are still obscure. In Drosophila larvae, rhodopsin acts as a thermosensor^24^. In mammalian sperm cells, not only rhodopsin but also other opsins were shown to be involved in thermosensing^4^. Thus, a number of different opsins were demonstrated to be present in sperm cells, inhibition of specific opsins significantly reduced the thermotactic response, and sperm thermotaxis of rhodopsin-knockout mice was 70% reduced. This reduction was larger than expected for the elimination of a single thermosensor out of several, suggesting the importance of rhodopsin for thermotaxis and possibly the existence of a lattice-like array of thermosensors that is disrupted when rhodopsin is removed^4^. Such an array of rhodopsin is known to be present in the visual system^25^. Notably, the signaling pathways known to function in the visual system, the phospholipase C (PLC) pathway^26^ and the transducin/cyclic nucleotide pathway^27^, also function in sperm thermotaxis^2,4^. In the visual system, the opsin involved in the PLC pathway is melanopsin^26,28^, whereas the other opsins are likely involved in the transducin/cyclic nucleotide pathway^27,29,30^. In sperm cells, inhibition of either pathway alone causes partial inhibition of thermotaxis, but inhibition of both together completely inhibits thermotaxis^4^. All these suggest that both rhodopsin and melanopsin may be important for sperm thermotaxis.

Mammalian sperm cells are able to respond to extraordinarily small temperature differences. For example, a human sperm cell is able to thermotactically respond to a temperature difference as small as <0.0006 °C when it swims its body-length distance, and do it over a wide temperature range (at least 29–41 °C)^3^. To explain this hypersensitivity, it was speculated that each sperm cell contains multiple opsin types, each type differently distributed in the cell and associated with only one of the two signaling pathways^4^. Here we put these speculations to the test, employing rhodopsin and melanopsin as test cases.

## Results

**Figure 1:**
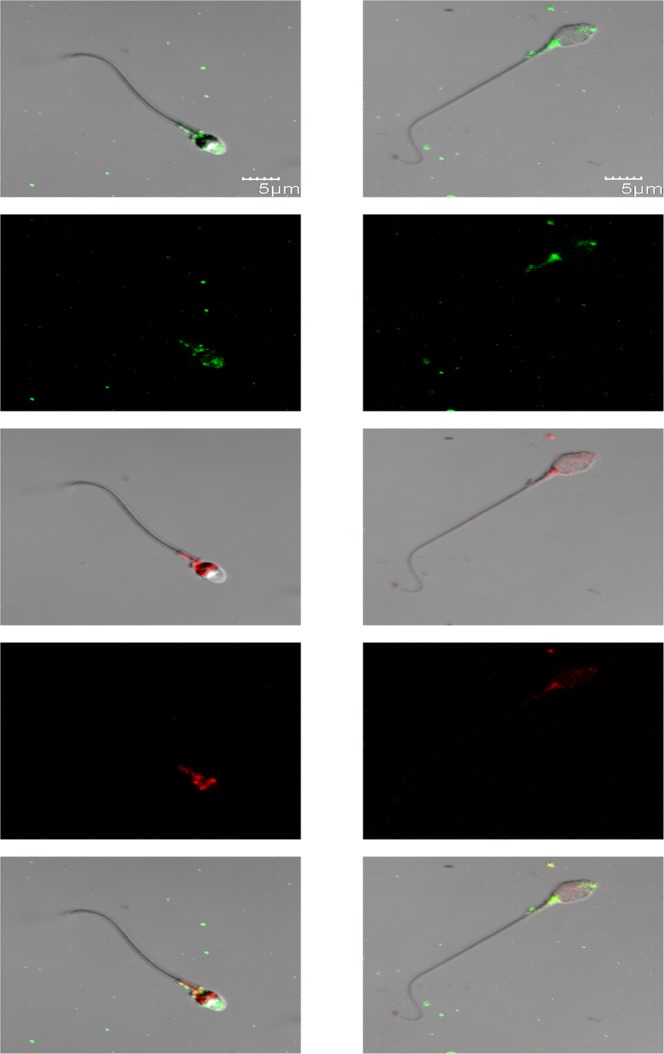
Representative confocal images (average of four Z projected slices) showing the locations of rhodopsin and melanopsin in two human sperm cells, revealed by immunocytochemical analysis. Rhodopsin and melanopsin are stained in green and red, respectively. See Methods for the antibodies used. Each column shows one of the cells at the following five configurations (from top to bottom): rhodopsin staining overlaid with the DIC image of the cell, rhodopsin staining, melanopsin staining overlaid with the DIC image of the cell, melanopsin staining, and both rhodopsin and melanopsin staining overlaid with the DIC image of the cell.

**Figure 2:**
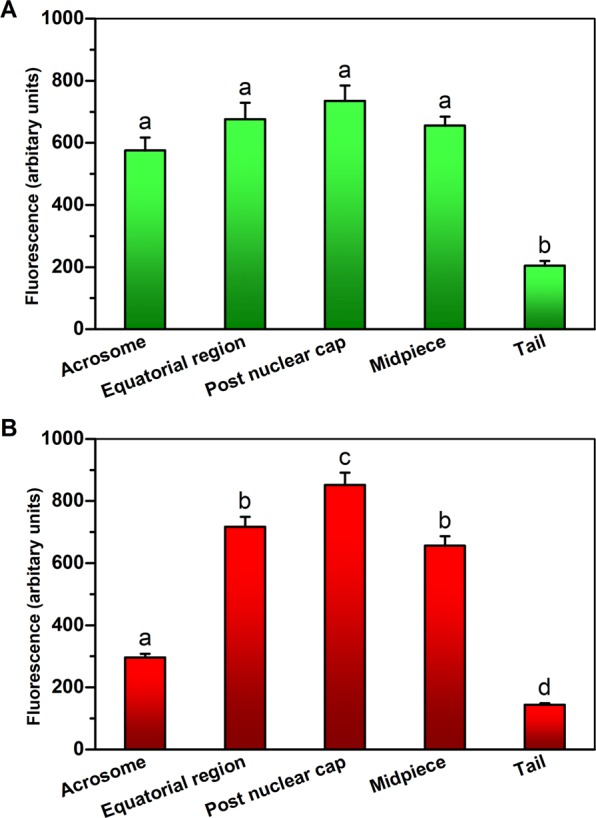
Presence of rhodopsin and melanopsin in different regions of human sperm cells. The figure was drawn from the data of individual cells shown in Table S1. The values shown are mean ± SEM of 6 sperm samples from different donors. (**A**) Rhodopsin. (**B**) Melanopsin. Columns marked with different letters were significantly different (*P* < 0.001 for rhodopsin and *P* < 0.01 for melanopsin) according to repeated measures ANOVA with Tukey–Kramer post-test (n = 60–154 cells for rhodopsin and 188–209 for melanopsin).

**Figure 3:**
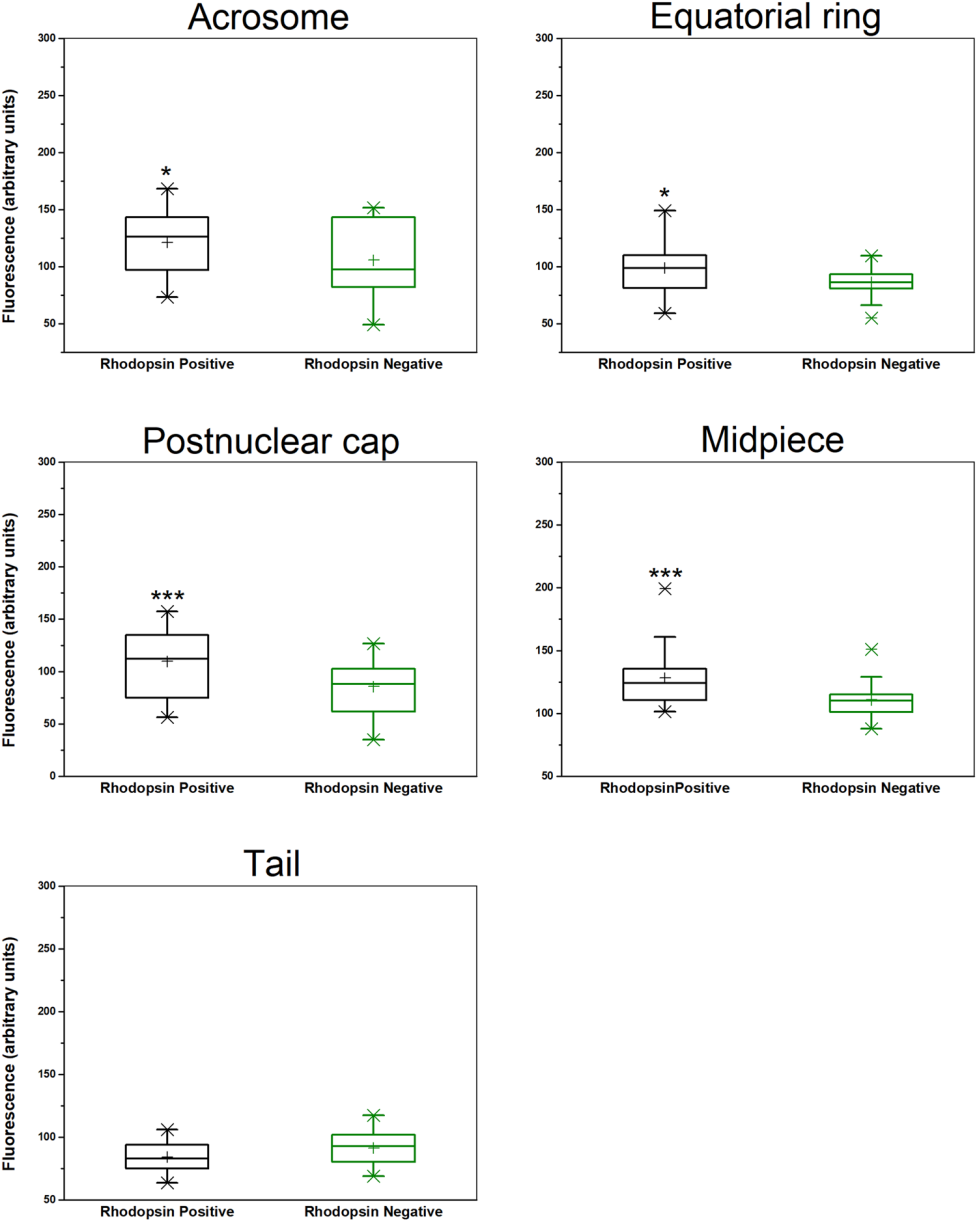
Comparative analysis of rhodopsin staining in wild-type and rhodopsin-null mice. Sperm cells were retrieved from C57BL/6 wild-type and C57BL/6 Rho^−/−^ knockout mice^42^. *P < 0.05 and ***P ≤ 0.005 according to two-tailed Mann-Whitney Test (n = 27 and 33 cells for rhodopsin-positive and negative, respectively). The higher and lower boundaries of each box indicate the 75th and 25th percentiles, respectively; the line within the box marks the median; the plus sign marks the mean. Whiskers above and below the box indicate the 95th and 5th percentiles, respectively. X denotes an experimental point outside the 5th–95th percentiles.

**Figure 4:**
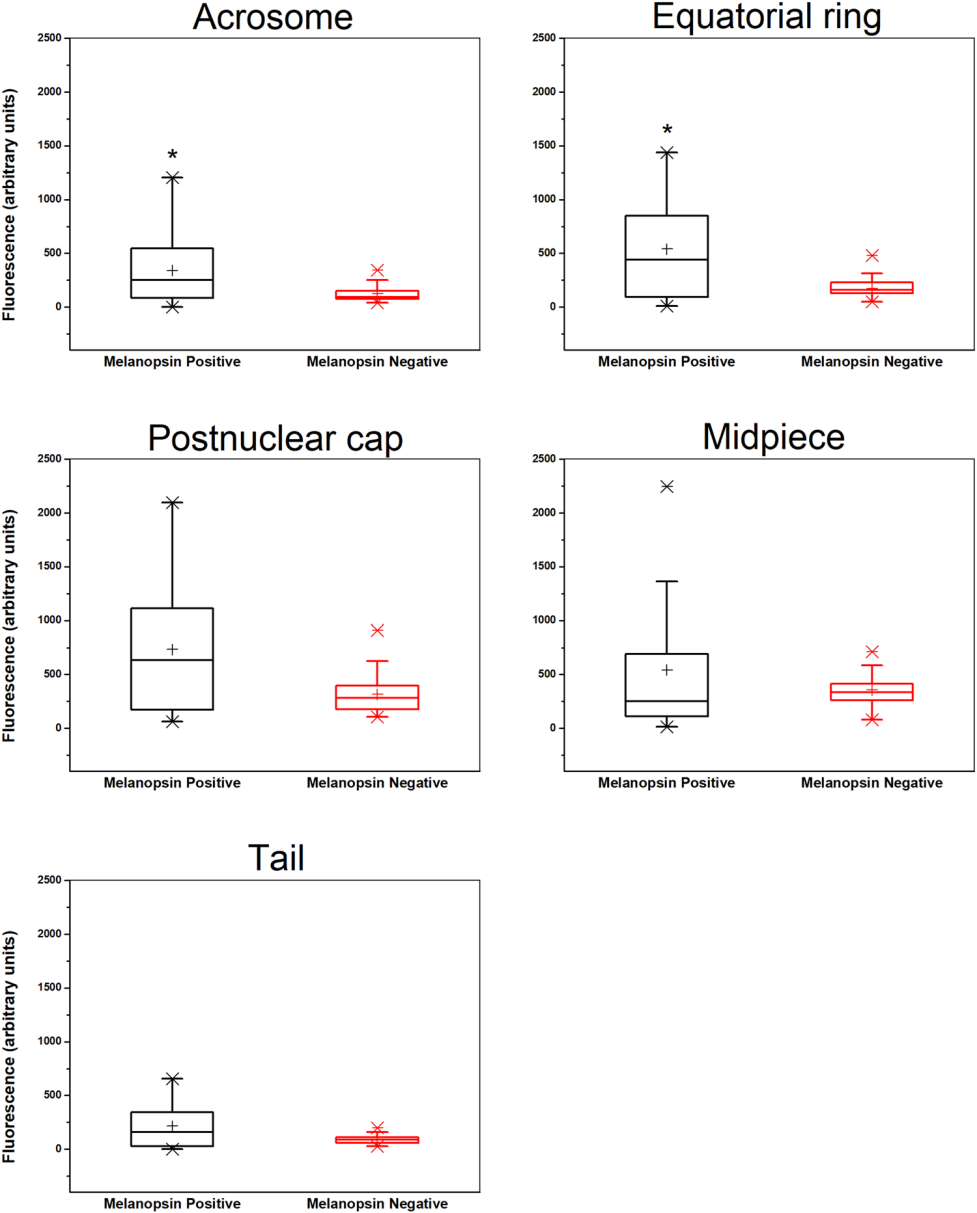
Comparative analysis of melanopsin staining in wild-type and melanopsin-null mice. Sperm cells were retrieved from C57BL/6 wild-type and *Opn4*^−/−^*; Opn4:tdTomato*^+^ mice. *P < 0.03 according to two-tailed Mann-Whitney Test (n = 16 and 38 cells for melanopsin-positive and negative, respectively). See Fig. 3 for details.

To determine whether each sperm cell contains both rhodopsin and melanopsin, which, as summarized above, are exclusively located within a different cell type in the visual system, we took an immunocytochemical approach. We incubated human sperm cells, which had been allowed to capacitate, with goat anti-rhodopsin antibody and, simultaneously, with rabbit anti-melanopsin antibody, and then with specific fluorescent secondary antibodies. As a control for the specificity of the secondary antibodies, we skipped the incubation with the primary antibodies and incubated the cells with the fluorescent secondary antibodies only. Employing confocal microscopy, we found that the majority of sperm cells were indeed stained by both antibodies (Table 1), indicating that most of the cells contained both opsins together. The antibodies primarily stained the postnuclear cap, midpiece, equatorial ring and, in the case of rhodopsin, the acrosome, but other locations were occasionally stained as well (Figs. 1, 2; see Supplementary Fig. S1 and Table S1 for detailed analysis). As a control for the specificity of the primary antibodies, we also carried out these experiments with mouse sperm cells, comparing between wild-type and rhodopsin- or melanopsin-knockout mice. A quantitative analysis revealed specific staining with anti-rhodopsin antibody in the acrosome, equatorial ring, postnuclear cap and midpiece (Fig. 3) and with anti-melanopsin antibody in the acrosome and the equatorial ring (Fig. 4). While we cannot validate, in the absence of a similar negative control for human sperm cells, the locations of these opsins in human cells, the results, combined together, suggest that rhodopsin and melanopsin are present at several locations in the cell.

**Table 1:** Both rhodopsin and melanopsin are present in same sperm cells.

Types of stained cells	Percentage of cells^a^
Cells stained by both anti-rhodopsin and anti-melanopsin antibodies^b^	81
Cells stained by anti-rhodopsin antibody only	0
Cells stained by anti-melanopsin antibody only	19
Cells neither stained by anti-rhodopsin nor by anti-melanopsin antibodies	0

Human sperm cells were washed and incubated for capacitation as described in Methods.

^a^The total number of cells used for the counting and considered as 100% was 58 (out a total of 105 cells photographed; staining of the other cells could not be determined with sufficient confidence).

^b^See Methods for the antibodies used. Cells were considered as stained when the measured intensity of their staining per unit area was both larger than the sum mean + SD of the negative control (staining with secondary antibody only) and larger than 150% of the mean of the negative control.

**Figure 5:**
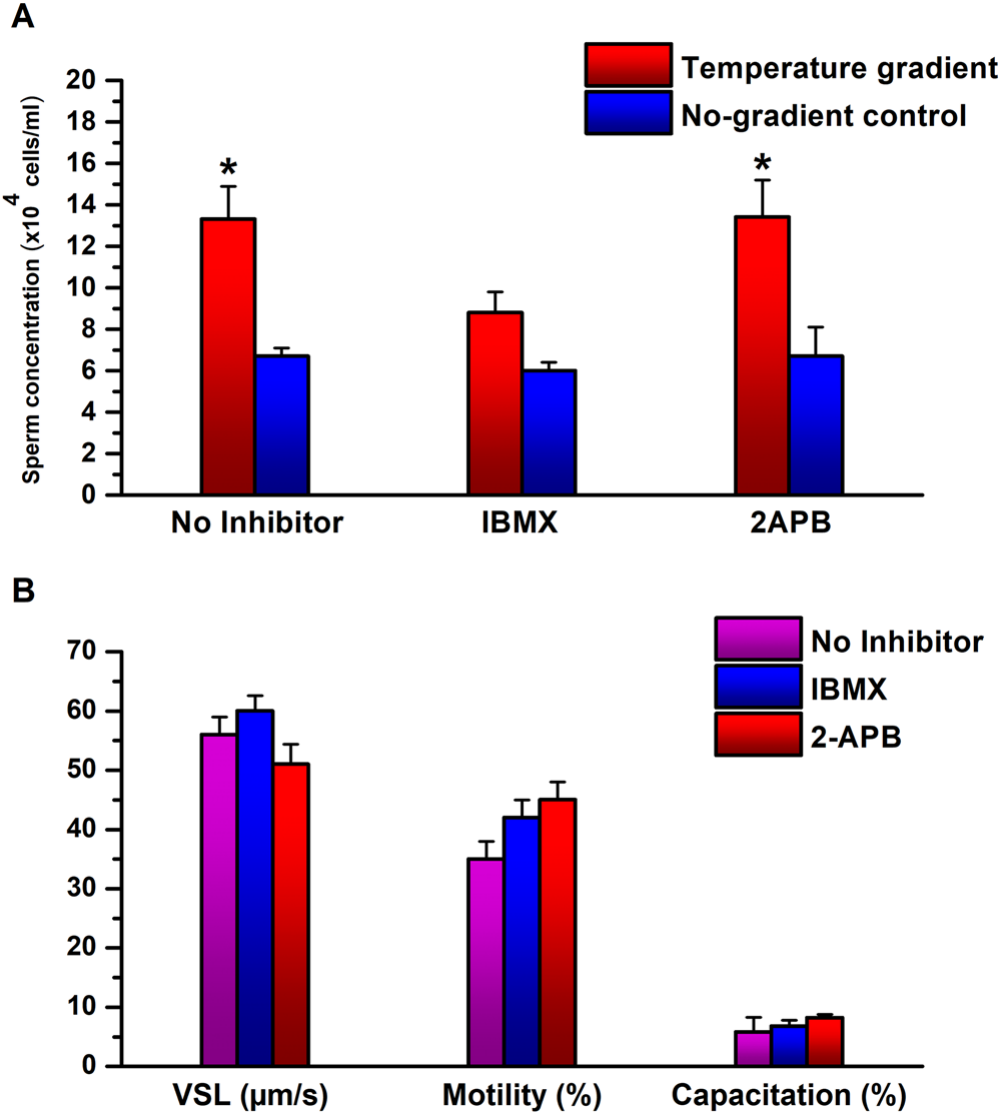
Effects of inhibitors of the transducin/cyclic nucleotide pathway and of the PLC pathway on sperm cells of mice that do not express melanopsin. (**A**) Effects on sperm thermotaxis. The columns stand for the accumulation of sperm cells in the warmer compartment, using the two-compartment separation tube (mean ± SEM of 6 *Opn4*^−/−^*; Opn4:tdTomato*^+^ mice). All values were normalized according to the average number of sperm cells in the cold chamber (6.6 × 10^6^ cells). *P < 0.01 for the difference between gradient and no-gradient control according to repeated measures ANOVA with Tukey–Kramer post-test. (**B**) Effects on sperm capacitation and motility parameters. The values shown are of the very same sperm samples used in A. Note that the same scale applies to the velocity and percentages. None of the values was significantly different from the no-inhibitor control according to repeated measures ANOVA with Tukey–Kramer post-test. See Table S2 for all measured motility parameters.

In vision, rhodopsin activates the transducin/cyclic nucleotide signaling pathway^27^ and melanopsin triggers the PLC pathway^26^. To put to the test the speculation that this is also the case in sperm thermotaxis^4^, we measured thermotaxis of sperm cells retrieved from mice that do not express melanopsin (*Opn4*^−/−^; *Opn4:tdTomato*^+^). These mice are expected to signal in thermotaxis via the transducin/cyclic nucleotide pathway only. This is because, in vertebrate vision, only melanopsin signals via the PLC pathway^31^. Our expectation was that thermotaxis of sperm cells from these mice should be sensitive to an inhibitor of the transducin/cyclic nucleotide pathway but not to that of the PLC pathway. We, therefore, tested the effects of inhibitors of these pathways on sperm thermotaxis of these mice. We compared the effects of 3-isobutyl-1-methylxanthine (IBMX), a phosphodiesterase inhibitor that inhibits the transducin/cyclic nucleotide pathway^32^, with that of 2-aminoethoxydiphenyl borate (2APB), a PLC pathway inhibitor^33^. IBMX was shown earlier to have comparable inhibitory effects on sperm thermotaxis as do other phosphodiesterase inhibitors, and to be highly effective^4^. 2APB blocks both store-operated Ca^2+^ channels (SOCs) and IP_3_R, but SOCs are not involved in sperm thermotaxis, and 2APB partially inhibits thermotaxis by blocking the IP_3_R Ca^2+^ channel^2^. It was shown to be effective and comparable to another PLC inhibitor^2^. As expected, while IBMX significantly inhibited thermotaxis, 2APB (verified to be effective on wild-type sperm cells) did not (Fig. 5A). Other parameters that can affect sperm accumulation in the warmer compartment — the fraction of motile cells, their linear velocity, and the level of capacitated cells (defined herein as the level of A23187-induced acrosome-reacted cells^9,34^) — did not contribute to the inhibition by IBMX and were not affected by the inhibitors (Fig. 5B). The lack of effect of the inhibitors on motility was also evident from the no-gradient control, i.e., from the sperm migration to the other thermotaxis compartment in the absence of a temperature gradient, which was comparable in the presence and absence of the inhibitors (Fig. 5A), and from the other measured motility parameters (Table S2). Note that because capacitation reflects the sperm ability to fertilize an oocyte^7^ and because it involves many biochemical and signaling processes^35^, its level is an excellent control for the overall physiological state of the sperm cells. The comparable capacitation level between all three conditions tested (Fig. 5B) therefore suggests that the inhibitors did not affect the physiological state of the sperm cells. Taken together, the results suggest that in sperm cells, as in vision, rhodopsin and melanopsin each triggers a different signaling pathway.

**Figure 6:**
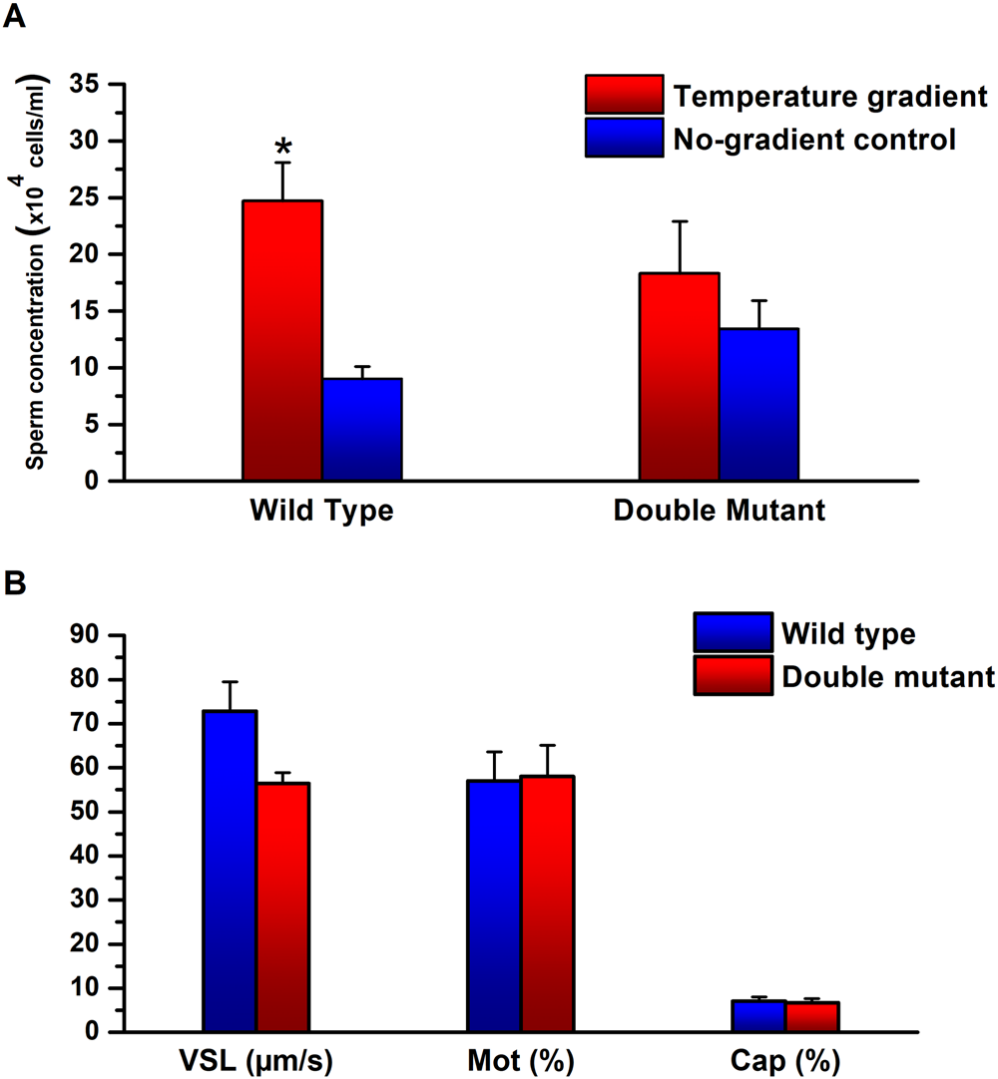
Activities of sperm cells retrieved from double-mutant mice lacking both rhodopsin and melanopsin. (**A**) Sperm thermotaxis. The columns stand for the accumulation of sperm cells in the warmer compartment, using the two-compartment separation tube (mean ± SEM of 11 *Opn2*^−/−^*; Opn4*^−/−^*; Opn4:tdTomato*^+^ mice). All values were normalized according to the average number of sperm cells in the cold chamber (11.4 × 10^6^ cells). *P < 0.05 for the difference between gradient and no-gradient control according to repeated measures ANOVA with Tukey–Kramer post-test. (**B**) Sperm capacitation and motility parameters. The values shown are of the very same sperm samples used in A. None of the values was significantly different from the no-inhibitor control according to repeated measures ANOVA with Tukey–Kramer post-test. See Table S3 for all measured motility parameters.

If this conclusion is correct, one would expect to have a much reduced thermotactic activity in sperm cells of double mutant mice lacking both rhodopsin and melanopsin proteins. (Some activity should be retained in such mice due to the presence of the other opsins). To examine this anticipation we studied the thermotactic activity of sperm cells retrieved from *Opn2*^−/−^*; Opn4*^−/−^*; Opn4:tdTomato*^+^ mice. Clearly, the thermotactic activity of this double mutant, if existed, was very low (Fig. 6A). The fraction of motile cells, their linear velocity, and the level of capacitated cells were not affected by the mutations (Fig. 6B). These results are consistent with the almost complete inhibition of sperm thermotaxis of wild-type mice by both IBMX and 2APB together^4^.

## Discussion

In this study we demonstrated that most sperm cells contain both rhodopsin and melanopsin. At least in the case of mouse sperm cells and likely also in human cells, each of these opsins appears to be present at several locations in the cell. These make a larger number of locations than found earlier by regular fluorescence microscopy^4^, probably due to the higher sensitivity of confocal microscopy. This study also provided evidence for the association of each of these opsin types with a different signaling pathway.

The earlier finding of opsins in mammalian sperm cells^4^, their involvement in thermotaxis^4^, and the temperature hypersensitivity of sperm cells for thermotaxis^3^ raised the possibility that each sperm cell may contain multiple opsin types, each type differently distributed in the cell and associated with only one of the two signaling pathways, and that these together contribute to the temperature hypersensitivity^4^. The results of this study thus appear to validate, with respect to rhodopsin and melanopsin, the multiplicity of opsin types in individual sperm cells and the association of each with a different signaling pathway. Yet, the results could not confirm the assumption of a different distribution of each opsin type, at least not major differences.

A comparison between the thermotactic activity of melanopsin-knockout mice (Fig. 5A) relative to wild-type mice (Fig. 6A) suggests that the absence of melanopsin caused ~50% decrease in thermotactic activity. This decrease seems larger than expected from knockout of a single opsin out of several. The same was argued for rhodopsin-knockout mice, where the thermotactic activity was found to be 70% reduced^4^. To account for the larger-than-expected reduction in thermotactic activity, it was proposed that rhodopsin is arranged in sperm cells in a lattice-like array (consistent with the high supramolecular organization of rhodopsin in the rod membrane in the retina^25^ — organization that appears essential for the photosensitivity of rod cells^36^), and this array is disrupted when rhodopsin is removed^4^. The same interpretation may hold for the larger-than-expected reduction in thermotactic activity in melanopsin-null mice, suggesting that both rhodopsin and melanopsin are part of the lattice, if indeed exists. Notably, the remaining ~50% activity in the case of melanopsin-null mice still enabled to measure the effect of inhibitors on the activity, unlike in the case of rhodopsin-null mice, where the remaining 30% activity was too low for testing it.

In conclusion, rhodopsin and melanopsin coexist in the same sperm cells, their distributions vary to some extent but they do co-localize, and, as in vision, each of them triggers a different signaling pathway: rhodopsin prompts the transducin/cyclic nucleotide signaling pathway and melanopsin — the PLC pathway. More studies are required to establish how these features contribute, if at all, to the extraordinary temperature sensitivity of mammalian sperm thermotaxis.

## Methods

### Antibodies

The anti-rhodopsin antibody (I-17) and anti-melanopsin antibody were from Santa Cruz Biotechnology (Heidelberg, Germany) and Novus Biologicals (Colorado, USA), respectively. The fluorescent secondary antibodies Alexa Flour® 488 Donkey anti-goat and Cy^TM^3-conjugated AffiniPure goat anti-rabbit were from Abcam (Berlin, Germany) and Jackson ImmunoResearch (Cambridgeshire, UK), respectively.

### Chemicals and media

IBMX, 2APB, poly-L-lysine, fluorescein isothiocyanate-Pisum sativum agglutinin (FITC-PSA), A23187, mineral oil and Fluoroshield mounting medium were procured from Sigma-Aldrich (Rehovot, Israel). Flushing Medium and Human tubal fluid medium (HTF) were purchased from Origio (Måløv, Denmark) and Irvine Scientific, (Santa Ana, CA, USA), respectively.

### Knockout mice lines

The *Opn4*^−/−^*; Opn4:tdTomato*^+^ mice^37^ and the *Opn2*^−/−^*; Opn4*^−/−^*; Opn4:tdTomato*^+^ mice^29,30^ were a generous gift from King-Wai Yau and Daniel Silverman from Johns Hopkins University School of Medicine (Baltimore, MD), and C57BL/6 Rho^−/−^ mice from Marian M. Humphries from Trinity College (Dublin, Ireland).

### Human sperm handling and capacitation

Studies with human sperm cells were approved by the Bioethics and Embryonic Stem Cell Research Oversight Committee of the Weizmann Institute of Science. The methods were carried out in accordance with the approved guidelines. Six human semen samples were obtained from healthy different donors after 3 days of sexual abstinence. Informed consent was obtained from each donor. Semen samples with normal sperm density, motility, and morphology (according to WHO guidelines^38^) were allowed to liquefy for 30–60 min at room temperature. For thermoseparation and for immunocytochemical analysis, human semen was mixed 1:1 with Flushing Medium (which includes human serum albumin, HEPES, bicarbonate, glucose, pyruvate and additional salts) and then separated from the seminal plasma and washed twice in capacitating medium (Flushing Medium supplemented with additional human serum albumin to a final concentration of 0.3%) by centrifugation (120 × g, 10 min). Subsequently, the sperm concentration was adjusted to 70 × 10^6^ cells/ml in the capacitating medium and incubated for 2 h under an atmosphere of 5% CO_2_ at 37 °C for capacitation^9^.

### Mouse sperm handling and treatments

Studies with mice were approved by the Institutional Animal Care and Use Committee of the Weizmann Institute of Science. The methods were carried out in accordance with the approved guidelines. Mice were sacrificed by cervical dislocation. Sperm cells were collected from the cauda epididymis of 3–5 month-old C57BL/6 wild-type (N = 11), C57BL/6 Opn4^−/−^*; Opn4:tdTomato*^+^ (N = 6), C57BL/6 *Opn2*^−/−^; *Opn4*^−/−^*; Opn4:tdTomato*^+^ (N = 11) and C57BL/6 Rho^−/−^ (N = 3) mice, and suspended in a droplet of HTF containing 1% BSA under mineral oil. Subsequently, the samples were incubated for 1 h under an atmosphere of 5% CO_2_ at 37 °C for capacitation. Incubations with inhibitors (IBMX, 2APB) were performed after capacitation. Sperm samples were incubated with each inhibitor under an atmosphere of 5% CO_2_ at 37 °C for 5 min prior to thermotaxis or motility assays. Stock IBMX and 2APB were dissolved in DMSO. In the negative controls, DMSO was added to a final concentration of 1%, like its concentration in the samples treated with the inhibitors. After each treatment the pH was confirmed to be 7.5. Sperm handling was under dim light.

### Thermotaxis assays

For thermotaxis assays and thermoseparation, the equally long two- (for mouse sperm cells) and three-compartment (for human sperm cells) thermoseparation tubes (internal diameter 3.8 and 4.1 mm, respectively) were filled with sperm cells as described^4^. The tube was placed in the thermoseparation device^2^, with the edge of the sperm-filled compartment at 35 °C and the other edge at 37 °C, creating a linear temperature gradient between these points, verified experimentally^3^. Following a 15- or 20-min separation period (for two or three compartments, respectively), the sperm cells were collected from the warmer compartment and counted using a haemocytometer or a Makler chamber. For the no-gradient control, the tube was placed in an incubator prewarmed at 35 °C.

### Motility analyses

Following the various treatments, sperm cells were diluted to 5 × 10^6^ cells/ml in HTF containing 1% BSA and the same concentration of inhibitor used for the treatment. For motility recordings, sperm cells were placed in a prewarmed Makler chamber over a 38 °C Thermo Plate (Tokai Hit, Shizuoka-ken, Japan). Short videos (10–15 s each) were made using a phase-contrast Nikon Alphaphot microscope equipped with a digital camera (u-Eye, Obersulm, Germany) at 75 frames/s. The analysis was carried out by both a homemade script for MatLab software and ImageJ freeware including CASA plug-in. The conditions for motion analysis followed the guidelines for CASA instruments^39^.

### Determination of the fraction of capacitated cells

The fraction of capacitated sperm cells was calculated as the difference between the fraction of acrosome-reacted cells, measured with fluorescein isothiocyanate-*Pisum sativum* agglutinin, before and after stimulation with the Ca^2+^ ionophore A23187 (dissolved in DMSO) for 30 min under an atmosphere of 5% CO_2_ at 37 °C^33,40,41^. As a negative control, the cells were similarly treated with DMSO instead of A23187. The stained slides were observed under a Nikon eclipse Ti-S microscope with a Nikon S Fluor 40X/0.90 NA objective (Nikon Instruments, Amsterdam, The Netherlands).

### Immunocytochemistry

After predefined treatment, human sperm cells were washed three times in PBS by centrifugation (500 × g for 1 min), the concentration was adjusted to 2 × 10^6^ cells/ml, and the cells were smeared on a 18 × 18 coverslip coated with 0.01% (w/v) poly-L-lysine and air-dried. The coating was performed by gently spreading ~0.6 ml 0.01% (w/v) poly-L-lysine on the coverslip, 5 min incubation at room temperature, discarding excess solution and allowing to dry at room temperature overnight. Cells were fixed for 5 min with 500 μl paraformaldehyde (4% v/v in PBS), washed three times with PBS, blocked for 30 min with bovine fetal serum (10% v/v in PBS), and then treated with a mixture of goat anti-rhodopsin I-17 and rabbit anti-melanopsin antibodies, each diluted 1:200 in bovine fetal serum (5% v/v in PBST) overnight at 4 °C, in a platform rocker at 25 RPM. A negative control was similarly prepared but without one or both of these primary antibodies. Following three 5-min washes in PBS on the platform rocker at 25 RPM, the coverslips were treated with Alexa 488-conjugated Donkey anti-Goat antibody (1:10,000), washed again three times in PBS, blocked with 5% BSA in PBST, treated with Cy-3 conjugated goat anti-rabbit antibody (1:10,000), and washed three times with PBS. Finally, Fluoroshield mounting medium (20 µl) was placed on each coverslip, and the coverslip was carefully flipped upside down on another 24 × 40 microscope coverslip. The same protocol was used for mouse sperm cells, except that the paraformaldehyde fixation step was omitted (due to too strong autofluorescence in the presence of paraformaldehyde).

### Confocal microscopy

The samples were observed under an Olympus FlowView confocal fluorescence microscope (Olympus FV1000, 60x oil-immersion objective Olympus UPLSAPO - NA 1.35, Tokyo, Japan). The lasers used for excitation were 488 nm (emission range 501–546 nm) and 560 nm (emission range 575–675 nm) with 10% transmissivity and 2 airy units of confocal pinhole. Images were visualized with FV10–ASW 4.2a software (Olympus, Tokyo) and analyzed by ImageJ (Win-64 version 7.1) with Z Projection (Max Intensity) and background subtraction using rolling ball (radius = 50 pixels). Regions of interest (acrosome, equatorial ring, postnuclear cap, midpiece and tail) were manually defined by differential interference contrast (DIC) images, and the mean fluorescence intensity per area unit was measured in each of them.

### Statistical analysis

The data were statistically analyzed by GraphPad InStat version 6 (USA).

## Supplementary information

Supplementary information.

## Data Availability

Data are available upon request.
