# Supplementary material for: Rhodopsin and melanopsin coexist in mammalian sperm cells and activate different signaling pathways for thermotaxis
Source: Sci Rep. 2020 Jan 10;10:112. doi: 10.1038/s41598-019-56846-5 (PMC6954197; doi:10.1038/s41598-019-56846-5)
Supplement: Supplementary file 1 — Supplementary information. [file 41598_2019_56846_MOESM1_ESM.pdf]

Rhodopsin and melanopsin coexist in mammalian sperm cells and activate different signaling pathways for thermotaxis

Debarun Roy, Kohava Levi, Vladimir Kiss, Reinat Nevo and Michael Eisenbach

Supplementary Information

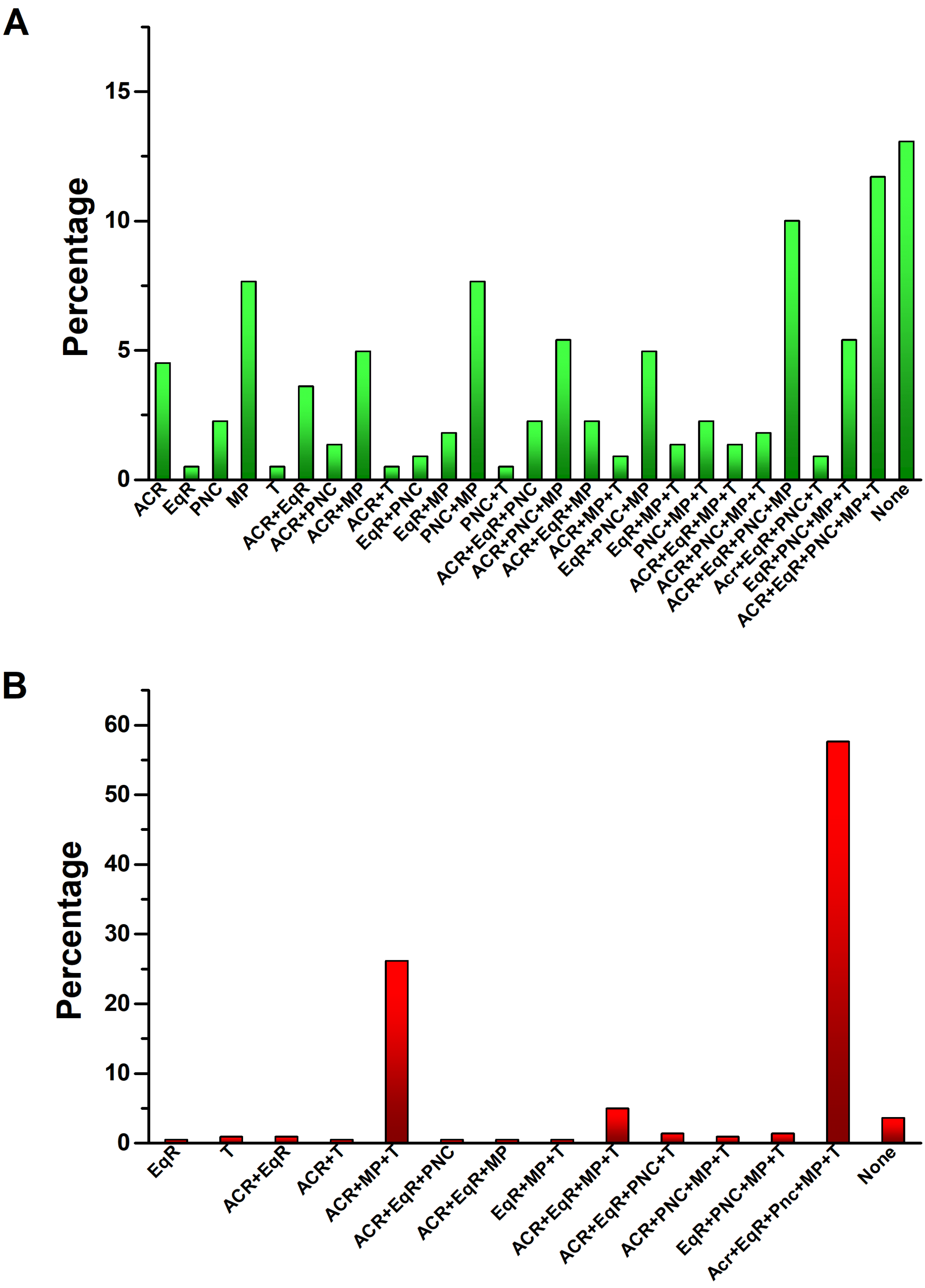

Figure S1. Distribution of rhodopsin and melanopsin in human sperm cells. The cells are those shown in Figure 2. The figure was drawn from the data of individual cells shown in Table S1. N is the number of cells analyzed. Abbreviations: Acr, acrosome; EqR, equatorial ring; PNC, postnuclear cap; MP, midpiece; T, tail. See Table S1 and Methods for more details.

	Rhodopsin					Melanopsin				
**Cell**	**Acr**	**EqR**	**PNC**	**MP**	**T**	**Acr**	**EqR**	**PNC**	**MP**	**T**
1	123	349	1630	1018	196	582	1684	2018	1745	243
2	164	452	1294	1310	143	581	2434	2647	1663	229
3	181	243	226	250	44	185	279	479	293	78
4	151	180	343	215	51	170	413	429	424	87
5	415	215	284	400	63	169	300	459	466	131
6	263	787	508	624	69	197	357	314	328	116
7	699	300	171	312	60	182	373	285	295	143
8	186	356	333	250	111	613	1983	1816	214	110
9	286	361	335	174	32	1079	1168	723	435	156
10	126	154	138	324	84	430	785	750	447	151
11	175	90	131	317	48	151	308	423	200	95
12	488	417	426	644	182	529	1271	1798	1302	327
13	762	661	3202	1629	189	967	1948	2951	1745	278
14	498	385	208	202	53	176	374	339	301	94
15	314	392	122	240	55	212	353	302	329	100
16	105	594	2954	1302	155	349	1913	2957	1735	282
17	940	882	457	758	102	340	434	314	296	72
18	1093	591	601	1126	63	505	446	427	468	83
19	222	374	276	917	167	201	381	319	258	121
20	160	296	229	206	112	401	1591	1156	184	105
21	239	308	379	200	50	206	246	330	236	69
22	78	233	174	214	57	454	1491	930	368	139
23	296	232	314	185	78	220	244	320	290	76
24	378	675	811	343	124	158	227	433	268	63
25	275	321	195	178	53	477	717	559	180	94
26	446	239	121	243	113	297	188	154	190	68
27	423	344	205	218	84	280	425	388	267	112
28	443	215	223	256	72	138	310	457	689	167
29	248	310	830	879	142	600	1554	2008	1418	208
30	379	213	166	255	70	203	209	207	229	57
31	86	141	120	236	44	191	304	369	499	86
32	199	210	116	256	55	238	356	403	472	84
33	124	224	171	334	39	139	310	426	372	82
34	216	246	395	356	66	388	1264	1319	667	146
35	59	76	83	136	40	200	419	432	138	82
36	330	315	391	628	251	306	302	300	314	122
37	732	3438	1188	192	59	238	720	394	163	97
38	235	255	264	403	63	176	550	504	535	111
39	402	243	130	359	106	550	1083	964	416	202
40	165	500	454	379	98	708	1294	1400	546	188
41	205	225	573	390	81	651	1299	1030	506	169

	Rhodopsin					Melanopsin				
**Cell**	**Acr**	**EqR**	**PNC**	**MP**	**T**	**Acr**	**EqR**	**PNC**	**MP**	**T**
42	331	222	193	300	69	376	1207	1323	570	159
43	635	620	1017	1214	150	571	1666	1795	1737	254
44	199	278	261	352	97	220	445	469	423	140
45	174	415	250	430	65	150	449	572	434	102
46	417	346	484	572	63	236	622	753	1068	101
47	191	780	344	215	53	637	1421	1185	367	151
48	249	252	185	386	66	181	443	452	344	101
49	89	274	167	278	38	196	394	414	233	76
50	335	338	241	413	72	431	1238	1550	682	200
51	197	301	276	250	112	538	2011	1547	207	103
52	472	246	152	239	50	154	381	337	261	114
53	166	227	307	260	71	437	1019	953	436	170
54	89	142	129	233	36	189	499	419	168	77
55	406	269	182	296	63	518	1161	1023	543	164
56	116	97	246	260	33	134	268	491	279	54
57	118	810	615	899	79	105	242	364	624	66
58	937	405	199	267	70	329	245	175	197	63
59	337	530	1385	566	194	471	580	752	930	387
60	204	438	132	397	131	140	990	359	764	92
61	632	334	245	387	114	619	543	476	519	276
62	685	1084	1230	113	13	284	296	296	25	6
63	1392	2122	2102	1679	768	438	1765	2097	1898	413
64	872	744	473	790	153	220	846	993	783	114
65	677	801	3116	782	165	514	532	1775	932	137
66	124	538	559	456	243	73	415	627	480	91
67	474	552	470	1085	187	506	603	1234	1323	444
68	146	343	335	300	60	167	745	377	291	67
69	73	137	207	261	73	70	397	511	528	67
70	249	304	118	240	59	137	354	283	259	91
71	229	941	590	850	167	115	975	994	1686	161
72	442	1113	2978	1073	244	157	396	790	475	201
73	239	376	148	173	47	69	198	128	135	38
74	68	248	369	177	44	79	498	272	370	62
75	168	177	159	211	76	320	252	227	220	115
76	397	570	318	528	172	299	764	1543	990	120
77	325	199	456	703	140	645	458	871	660	306
78	353	74	97	242	92	256	169	302	364	234
79	474	2843	1049	1918	86	220	738	409	470	68
80	164	727	1630	188	74	148	946	447	248	97
81	607	319	629	810	232	412	559	1460	830	319
82	170	171	454	548	148	368	522	782	629	266

	Rhodopsin					Melanopsin				
**Cell**	**Acr**	**EqR**	**PNC**	**MP**	**T**	**Acr**	**EqR**	**PNC**	**MP**	**T**
83	146	300	472	1021	72	133	526	793	1551	109
84	107	143	119	129	69	387	393	411	283	151
85	163	144	152	364	97	480	367	404	344	224
86	558	1468	870	884	368	222	934	787	369	197
87	280	286	450	727	112	330	690	1121	1781	132
88	399	885	720	1221	392	199	623	988	1340	241
89	246	167	160	235	94	289	272	378	388	207
90	215	188	133	352	68	307	464	451	337	152
91	1616	1282	1681	1125	104	709	1609	1848	1656	93
92	361	1228	730	1167	363	175	978	910	1250	209
93	578	1016	952	1145	759	264	1465	1169	1463	291
94	408	731	378	309	79	211	831	830	847	103
95	2808	2806	2210	720	105	821	1203	746	245	66
96	1117	404	75	109	64	294	385	248	132	43
97	836	567	735	740	148	380	524	1046	930	121
98	667	493	377	577	61	441	767	1139	1283	100
99	634	459	247	275	100	163	540	269	525	82
100	2503	1384	985	1584	216	527	798	566	1808	266
101	79	214	150	347	65	129	521	686	936	120
102	608	354	551	424	118	289	460	736	581	69
103	95	223	582	118	47	194	331	246	105	48
104	363	548	201	409	78	362	981	1070	1053	181
105	1328	599	1038	2045	64	413	843	803	1276	91
106	401	679	810	465	43	340	901	965	1085	104
107	602	858	277	539	59	325	498	545	719	59
108	152	352	376	248	47	140	441	210	266	41
109	575	341	674	273	46	298	526	1000	873	78
110	578	362	387	341	62	207	904	702	480	111
111	416	582	571	527	92	171	404	391	507	145
112	139	225	350	258	130	385	868	998	235	181
113	278	370	1221	732	100	220	412	1837	1205	232
114	223	300	455	498	145	485	902	821	538	356
115	455	250	560	434	70	281	564	579	461	125
116	233	312	401	317	79	180	657	651	519	128
117	34	82	41	115	25	32	42	39	40	44
118	200	337	789	930	190	549	1820	1797	1134	206
119	267	383	358	230	70	209	565	772	399	97
120	277	261	424	438	86	171	457	620	451	139
121	217	338	752	545	90	197	504	476	346	116
122	197	392	470	1131	141	571	1771	1228	1486	193
123	255	433	1257	1607	172	504	1542	3129	2105	234

	Rhodopsin					Melanopsin				
**Cell**	**Acr**	**EqR**	**PNC**	**MP**	**T**	**Acr**	**EqR**	**PNC**	**MP**	**T**
124	321	272	329	408	87	193	568	596	606	191
125	194	400	1457	1477	255	548	1487	1749	1244	256
126	99	286	325	299	60	142	392	573	486	101
127	149	147	155	279	50	116	244	469	354	114
128	230	356	384	334	30	309	426	454	431	82
129	187	216	150	330	41	215	547	408	289	88
130	340	331	554	387	76	164	448	579	450	121
131	179	220	556	1496	159	432	1813	1734	1570	216
132	475	302	504	493	68	230	530	604	521	125
133	255	416	323	307	77	197	829	667	501	130
134	117	151	298	657	59	80	111	171	154	55
135	166	292	351	626	162	329	742	1126	493	325
136	138	255	259	341	127	312	799	1171	455	313
137	260	183	318	668	118	265	743	769	310	255
138	145	317	541	350	76	155	590	648	473	121
139	341	178	249	539	79	368	230	229	248	88
140	103	133	120	77	33	51	93	82	66	46
141	149	446	477	574	86	145	585	1020	1090	120
142	179	253	281	617	126	165	543	504	542	147
143	295	271	383	715	94	134	703	709	713	139
144	260	460	521	480	133	266	949	1402	1045	193
145	171	187	168	734	134	104	127	118	278	73
146	415	290	332	241	78	138	296	860	570	114
147	324	292	979	381	55	197	1135	875	1017	95
148	40	183	30	61	28	30	49	44	33	26
149	78	236	753	312	33	66	362	203	124	20
150	198	246	498	380	112	311	835	905	283	161
151	388	270	517	635	74	125	409	504	505	128
152	431	783	228	289	116	212	1130	453	629	119
153	235	293	436	427	93	148	483	513	471	199
154	136	165	234	616	92	150	169	198	278	95
155	78	343	448	507	91	129	723	1123	1035	141
156	143	230	196	213	65	144	350	291	210	85
157	605	520	277	360	56	241	588	521	400	86
158	250	266	399	787	70	164	458	592	794	117
159	54	125	122	311	89	43	64	67	66	66
160	121	379	824	493	73	160	535	1002	985	104
161	64	82	40	39	34	27	36	23	24	26
162	136	113	155	132	158	79	80	90	67	48
163	153	358	369	665	61	174	447	744	390	106
164	316	484	659	387	118	187	678	1237	569	143

	Rhodopsin					Melanopsin				
**Cell**	**Acr**	**EqR**	**PNC**	**MP**	**T**	**Acr**	**EqR**	**PNC**	**MP**	**T**
165	298	226	247	286	188	303	376	304	200	101
166	351	255	503	890	108	265	276	330	337	91
167	113	145	128	813	73	94	87	84	201	59
168	634	285	283	442	106	144	146	134	191	70
169	173	223	740	464	125	512	1346	1621	1030	231
170	232	296	160	728	37	134	400	509	682	76
171	255	266	249	401	49	213	314	798	689	116
172	55	117	35	63	32	61	69	44	40	36
173	180	203	560	579	83	190	440	859	993	117
174	201	194	183	346	94	225	331	392	528	140
175	168	395	244	358	159	331	873	790	418	218
176	99	390	366	544	121	149	850	801	1385	175
177	848	428	409	1265	240	363	1006	1234	627	357
178	157	350	477	375	89	224	1112	1668	652	142
179	428	190	235	678	111	258	231	238	307	110
180	210	629	699	624	126	200	1077	1450	1199	192
181	101	319	565	427	60	146	679	760	786	102
182	453	314	575	358	135	217	583	1483	730	230
183	224	212	352	452	55	161	312	499	615	118
184	162	226	541	394	66	234	324	531	578	139
185	190	483	635	1334	178	620	1877	1856	1477	274
186	80	397	244	485	40	83	747	620	738	87
187	197	182	171	160	88	204	321	281	216	79
188	189	213	214	189	80	230	323	375	236	83
189	452	381	1287	1094	264	629	1685	1848	1477	359
190	270	444	550	416	87	469	1437	1297	877	196
191	12	9	7	8	13	11	11	11	9	11
192	288	384	260	338	140	443	1141	813	384	287
193	677	503	561	377	136	339	960	1066	439	395
194	359	213	201	628	51	140	262	581	644	102
195	162	322	478	569	73	161	397	534	709	120
196	280	189	496	623	94	148	399	1158	965	157
197	381	312	326	561	59	175	411	658	502	110
198	225	384	1334	1438	303	666	1566	1894	2019	297
199	391	395	601	535	94	243	1275	1175	863	254
200	140	157	136	243	78	147	183	162	186	83
201	98	110	126	306	53	126	334	422	491	98
202	131	118	505	360	61	210	475	827	531	106
203	79	1148	218	319	74	146	486	441	427	89
204	333	483	290	229	60	319	570	699	280	94
205	142	187	99	262	58	123	417	491	329	94

	Rhodopsin					Melanopsin				
**Cell**	**Acr**	**EqR**	**PNC**	**MP**	**T**	**Acr**	**EqR**	**PNC**	**MP**	**T**
206	112	129	528	534	52	140	396	902	749	77
207	55	166	359	440	82	97	350	451	603	98
208	155	225	371	471	80	168	429	832	696	98
209	175	208	190	227	92	163	407	466	498	182
210	257	294	450	539	53	161	516	807	1250	90
211	2246	2333	1450	651	180	520	650	702	382	73
212	580	684	1197	614	246	328	330	402	183	109
213	353	757	887	368	131	144	256	474	283	62
214	1824	1085	379	1005	291	485	360	242	502	95
215	970	894	497	764	124	342	436	334	309	80
216	1074	598	611	1058	82	485	433	446	466	92
217	896	442	212	247	75	322	261	172	189	60
218	467	591	463	288	233	218	290	259	264	111
219	656	171	247	351	77	242	150	236	198	75
220	394	206	156	242	80	205	208	212	232	56
221	478	229	131	261	101	324	185	150	204	68
222	119	808	672	960	69	102	243	372	661	61

Table S1. Intensity of rhodopsin and melanopsin staining in different regions of individual human sperm cells. Each row represents an individual sperm cell. The values shown are the relative fluorescens intensities in the acrosome (Acr), equatorial ring (EqR), postnuclear cap (PNC), midpiece (MP) and tail (T). Fluorescens intensities of rhodopsin and melanopsin higher than the threshold values (staining per unit area larger than the sum mean+SD of the negative control and larger than 150% of the mean of the negative control) are highlighted in green and red, respectively. Cells were considered as stained when the measured intensity of their staining per unit area was both larger than the sum mean+SD of the negative control (staining with secondary antibody only) and larger than 150% of the mean of the negative control. Only highlighted values were considered as true staining. The values shown are mean of 6 sperm samples from different donors.

Animal	Treatment	VCL (µm/s)	VSL (µm/s)	VAP (µm/s)	LIN	WOB	Motility (%)
1	IBMX	219±15	57±10	86±8	0.68±0.09	0.41±0.05	46
	2-APB	247±26	41±8	70±8	0.57±0.08	0.29±0.02	39
	Control	233±22	50±9	71±5	0.71±0.11	0.32±0.04	36
2	IBMX	267±20	56±7	102±9	0.61±0.05	0.39±0.02	33
	2-APB	230±22	48±5	76±6	0.66±0.04	0.36±0.02	44
	Control	206±14	47±5	71±5	0.65±0.04	0.35±0.01	36
3	IBMX	217±18	56±7	86±7	0.63±0.05	0.44±0.03	38
	2-APB	271±12	46±4	94±4	0.51±0.03	0.36±0.01	54
	Control	187±14	52±5	71±5	0.72±0.03	0.40±0.01	24
4	IBMX	267±16	61±4	94±5	0.65±0.03	0.39±0.01	49
	2-APB	250±10	64±4	93±4	0.70±0.02	0.38±0.01	38
	Control	269±13	60±4	92±4	0.64±0.02	0.37±0.01	37
5	IBMX	284±11	71±4	110±5	0.65±0.02	0.40±0.01	42
	2-APB	269±9	59±3	106±4	0.59±0.02	0.41±0.01	50
	Control	250±14	66±7	107±7	0.60±0.04	0.45±0.02	42
6	IBMX	258±19	65±9	94±9	0.67±0.05	0.40±0.02	60
	2-APB	256±33	51±7	100±14	0.54±0.05	0.42±0.04	31
	Control	254±20	64±7	91±9	0.70±0.04	0.36±0.02	31

Table S2. Effects of inhibitors of the transducin/cyclic nucleotide pathway and of the PLC pathway on the motility parameters of sperm cells of mice that do not express melanopsin. Sperm samples from *Opn4*$^{-/-}$*; Opn4:tdTomato*$^{+}$ mice were subjected to IBMX (1 mM), 2APB (0.1 mM), or, as a control, the solvent DMSO, and then the motility was analyzed (37°C). Abbreviations: VSL, straight-line velocity (the time-average velocity of the sperm head along a straight line from its first position to its last position, expressed in µm/s); VCL, curvilinear velocity (time-averaged velocity of a sperm head along its actual curvilinear path, expressed in µm/s); VAP, average path velocity (velocity over an average path generated by a roaming average, expressed in µm/s), LIN, linearity [defined as (VSL/VCL)×100][1]; MOT (%), percentage of motile spermatozoa. The values are the mean ± SEM of the indicated 6 mice (~300 cells analyzed for each mouse). None of the parameters in the presence of an inhibitor was significantly different from the no-inhibitor control.

Animal	VCL (µm/s)	VSL (µm/s)	VAP (µm/s)	LIN	WOB	Motility (%)
Rho^{-} Mel^{-} (n=7)	269±9	56±2	92±4*	0.63±0.02	0.36±0.02*	46
Rho^{+} Mel^{+} (n=8)	266±9	73±7	120±3	0.62±0.04	0.45±0.01	33

Table S3. Motility parameters of sperm cells of mice that do not express both melanopsin and rhodopsin (*Opn2*$^{-/-}$*; Opn4*$^{-/-}$*; Opn4:tdTomato*$^{+}$) and of wild-type mice. The results are expressed as mean ± SEM. **P*<0.001 according to unpaired *t* test. See Table S2 for details.

References

1. Davis, R. O. & Siemers, R. J. Derivation and reliability of kinematic measures of sperm motion. *Reprod. Fertil. Dev.* **7,** 857–869 (1995).
